# Phenylalanine homeostasis in metabolic disorders: epidemiological trends, pathophysiological mechanisms, and clinical treatment

**DOI:** 10.3389/fendo.2026.1814249

**Published:** 2026-03-27

**Authors:** Yingting Chen, Shiqi Lu, Chaoqun Li, Yang Li, Chenge Qin, Yong He, Yanjun Niu, Qin Sun

**Affiliations:** 1 Department of Endocrinology and Metabolism, Jinhua Municipal Central Hospital, Jinhua, Zhejiang, China; 2 School of Exercise and Health, Shanghai University of Sport, Shanghai, China; 3 College of Physical Education and Sport Science, Qufu Normal University, Shandong, Qufu, China; 4 Center for The Genetics of Host Defense, University of Texas Southwestern Medical Center, Dallas, TX, United States; 5 Respiratory and Critical Care Medicine Department, Yangpu Hospital, Tongji University, Shanghai, China; 6 College of Physical Education and Health Science, Zhejiang Normal University, Zhejiang, Jinhua, China

**Keywords:** homeostasis, insulin resistance, metabolic diseases, phenylalanine, therapeutic strategies

## Abstract

Metabolic diseases, characterized by dysregulated energy homeostasis, represent a major global health challenge. While research has traditionally focused on glucose and lipid metabolism, emerging metabolomic and epidemiological evidence implicates circulating amino acid imbalances as a key factor for metabolic diseases. Phenylalanine (Phe) is an essential aromatic amino acid primarily metabolized by hepatic phenylalanine hydroxylase. Epidemiological investigation demonstrates that elevated plasma Phe is a significant risk factor for obesity, type 2 diabetes mellitus (T2DM) and cancer. Mechanistically, phenylalanyl-tRNA synthetase mediates phenylalanylation of insulin receptor β, subsequently inhibiting insulin signal transduction. Meanwhile, Phe and its catabolites (e.g., phenylpyruvate) impair mitochondrial function, induce oxidative stress, and inflammation, ultimately lead to insulin resistance and hepatic steatosis. Interestingly, the derivatives of Phe(such as exercise-induced N-lactoylphenylalanine) can suppress appetite and improve glucose homeostasis, suggesting functional diversity in the Phe metabolic network. In addition, clinical therapeutic strategies are gradually transitioning from traditional strict dietary restriction to personalized and multimodal interventions including nutrition, pharmacology and enzyme replacement therapy. However, ensuring the continuity, efficacy and safety of the treatment strategy remains a formidable challenge. In conclusion, this review explores the pathophysiological impact of Phe by integrating the epidemiological and molecular evidence for its role in metabolic diseases. From a translational medicine perspective, we further evaluate current therapeutic strategies, aiming to promote the clinical translation of Phe metabolism.

## 1 Introduction

Metabolic diseases, including obesity, type 2 diabetes mellitus (T2DM), and metabolic dysfunction-associated steatotic liver disease (MASLD), are primarily characterized by dysregulation of systemic energy homeostasis. The rising prevalence of these conditions imposes a major socioeconomic burden on global public health systems. For decades, research has centered on glucose and lipid metabolism disorders (e.g., insulin resistance and hyperlipidemia) as central drivers of metabolic diseases (1)However, emerging evidence highlights the role of circulating amino acid metabolism. Operating at the nexus of nutrient sensing, energy production, and signaling, amino acid dysregulation is now recognized as a key catalyst for obesity, diabetes, and their complications (2–6). For instance, high-throughput metabolomic studies utilizing liquid chromatography-mass spectrometry (LC-MS) have established that increased levels of specific plasma amino acids, particularly aromatic amino acids (AAAs), are effective biomarkers for early metabolic disease prediction (5, 7, 8).

As an essential AAA, phenylalanine (Phe) metabolism is predominantly governed by hepatic phenylalanine hydroxylase (PAH) (9). From the classical medical perspective, abnormal Phe metabolism is an important pathological basis of phenylketonuria (PKU). This inherited metabolic disorder, caused by mutations in the PAH gene, is defined by the biochemical hallmark of impaired conversion of Phe to tyrosine (Tyr), which results in systemic hyperphenylalaninemia (HPA) and subsequent severe neurotoxicity (9). However, current epidemiological research and multi-omics analyses reveal that such metabolic perturbations extend far beyond irreversible neurological impairment (including intellectual disability and epilepsy), and also gradually affect the peripheral metabolic health (9). Abnormally elevated plasma Phe levels have been identified as a significant risk factor for metabolic disorders (particularly T2DM) and have been implicated in tumorigenesis and cancer progression (5, 10, 11). Importantly, the functional paradigm of Phe has changed from a classical metabolic substrate to a pleiotropic signaling molecule. For instance, a recent large-scale study utilizing the French SNDS (Système National des Données de Santé) database reported that compared to healthy controls (n = 10,743), patients with PKU (n = 2,175) exhibited a significantly higher prevalence of hypercholesterolemia, diabetes, primary hypertension, and obesity (12). These findings suggest the systemic nature of Phe metabolic imbalance, extending beyond the central nervous system. Mechanistically, recent studies show that Phe directly inhibits insulin receptor β (IR-β) activity via phenylalanyl-tRNA synthetase (FARS)-mediated phenylalanylation, a novel post-translational modification, thereby inducing insulin resistance (13). Concurrently, downstream metabolites of Phe, such as phenylpyruvate, have been found to disrupt cellular energy homeostasis by inducing mitochondrial dysfunction (14, 15).

Interestingly, gut microbiota-derived Phe metabolites, such as phenylacetylglutamine (PAGln) and exercise-induced N-lactoyl-phenylalanine (Lac-Phe), demonstrate diverse and beneficial bioactivities in cardiovascular risk modulation and appetite regulation, respectively (16, 17). Consequently, modulating circulating Phe levels is important in explaining the pathogenesis of metabolic diseases and could serve as a therapeutic target for managing complications across multiple tissues and organs. Despite the availability of enzymatic therapies like Pegvaliase and pharmacological interventions such as Sapropterin, precisely targeting Phe and its derivatives to reverse chronic metabolic damage remains a formidable challenge (18–20).

Therefore, understanding the mechanisms by which dysregulated Phe triggers metabolic dysfunction is crucial. This review collects and analyzes the epidemiological and molecular evidence on the role of Phe in metabolic diseases, examines its pathophysiological impact, and evaluates current and emerging therapeutic strategies.

## 2 Natural sources and metabolism of phenylalanine

As an essential AAA, Phe possesses a carbon skeleton that cannot be synthesized endogenously, thus necessitating dietary intake to meet physiological requirements (21, 22). Phe is widespread in protein-rich foods, including animal-derived products (e.g., poultry, fish, eggs, dairy) and various plant-based sources like legume (23, 24). Upon entering the gastrointestinal tract, Phe is actively absorbed by the small intestinal epithelial cells into the portal circulation, followed by systemic distribution to various tissues for protein synthesis or entry into catabolic pathways (25, 26). Structurally, the core of the Phe molecule consists of a benzene ring linked to an α-amino acid backbone via a methylene bridge. This distinct aromatic configuration confers unique physicochemical properties that differentiate Phe from aliphatic amino acids, including pronounced hydrophobicity, the capacity for π-π stacking interactions, and its role as a primary substrate for the biosynthesis of diverse bioactive aromatic compounds (27).

**Figure 1:**
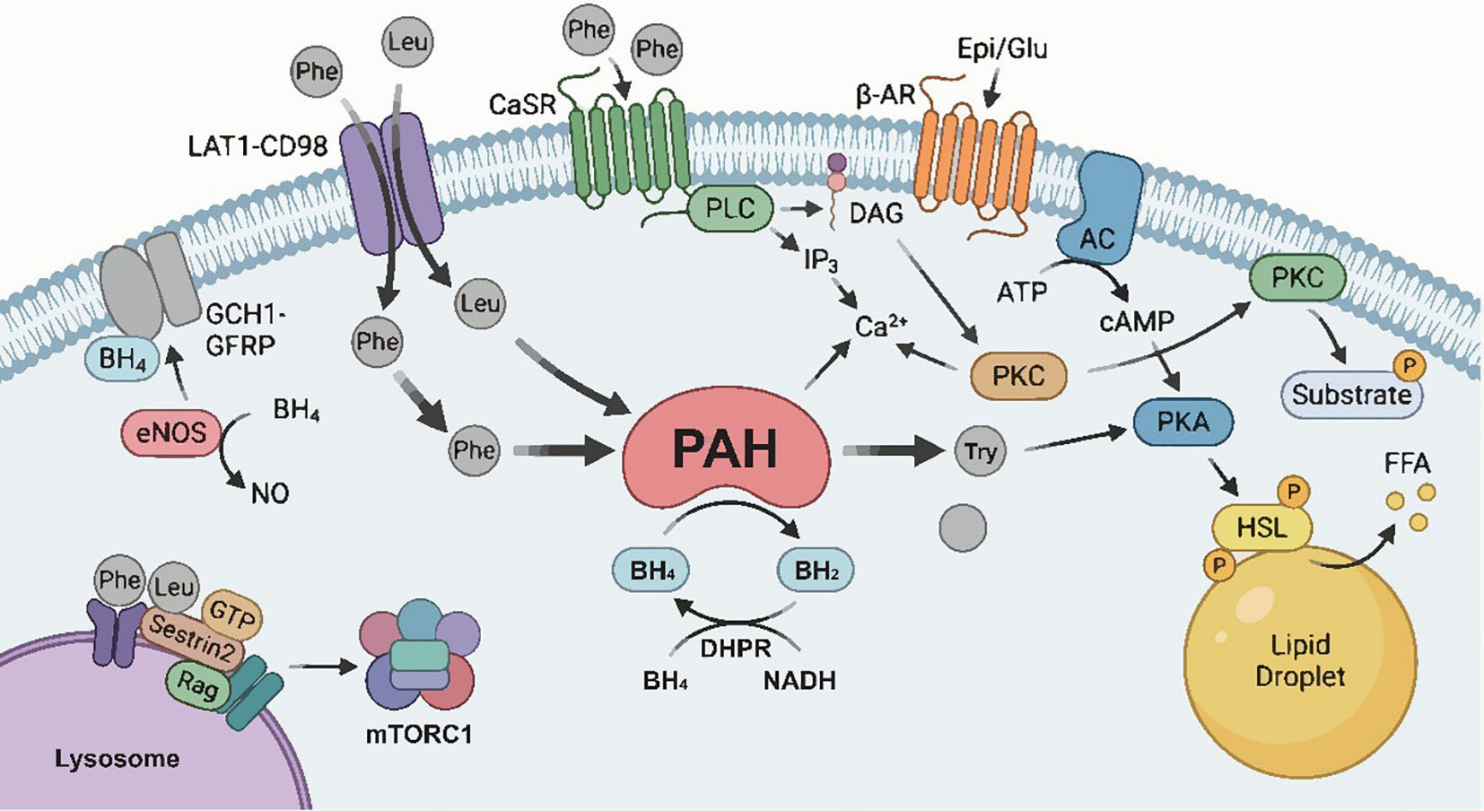
Phenylalanine as a metabolic signal integrator: coordination of nutrient sensing, hormonal regulation, and enzymatic activity.

Phe metabolism involves dynamic networks coordinated across multiple organs to maintain systemic homeostasis (**Figure 1**). Central to this process is the enzyme phenylalanine hydroxylase (PAH), a tetrahydrobiopterin-dependent monooxygenase primarily expressed in the periportal regions of the liver and the kidneys (28, 29). PAH maintains plasma Phe concentrations within a narrow physiological range (approximately 50–110 µmol/L) by catalyzing the irreversible hydroxylation of Phe into Tyr (28). Research indicates that kidney contribute approximately 60% of systemic Phe hydroxylation. Combined with splanchnic (primarily hepatic) activity, these organs account for nearly all whole-body hydroxylation flux (30, 31). Meanwhile, stable isotope tracer studies have demonstrated that the conversion of Phe via PAH provides approximately 10%–20% of the body’s endogenous Tyr supply (32). Tyr, in turn, serves as an indispensable precursor for the synthesis of thyroid hormones, melanin, and critical neurotransmitters, including dopamine, norepinephrine, and epinephrine (27). As a large neutral amino acid (LNAA), Phe transport across biological membranes relies on the L-type amino acid transporter 1 (LAT1), which functions as a heterodimer with the heavy chain glycoprotein CD98 (33, 34).Under physiological conditions, Phe competes with other AAAs (such as tryptophan and Tyr) and branched-chain amino acids (BCAAs, such as leucine and isoleucine) for the LAT1 channel (34, 35).

Due to the exceptionally high affinity of LAT1 for Phe (low K_m_ value), pathological elevations in blood Phe levels (as seen in PKU) lead to the saturation of LAT1 transport sites. This competitive inhibition precipitates a critical cerebral deficit of Tyr and tryptophan, which impairs the synthesis of essential neurotransmitters and impairs neurological integrity (34, 35).

Emerging evidence suggests that Phe acts not only as a metabolic substrate but also as a key signaling molecule that orchestrates cardiovascular function and metabolic homeostasis through direct or indirect regulatory mechanisms. For instance, Phe participates in classic substrate-driven negative feedback: when plasma Phe concentrations rise due to dietary intake or endogenous release, Phe acts as an activator of its own rate-limiting enzyme(PAH), thereby accelerating its metabolic conversion to Tyr (32). Similarly, Phe serves as an allosteric activator that directly binds to and activates the complex formed by GTP cyclohydrolase I (GCH1) and GCH1 feedback regulatory protein (GFRP) (36). This activation positively regulates the *de novo* synthesis of its cofactor tetrahydrobiopterin (BH_4_). As a requisite cofactor for endothelial nitric oxide synthase, BH_4_ is pivotal for maintaining nitric oxide bioavailability and vascular tone (36). Furthermore, cellular thermal shift assays have confirmed that exogenous Phe administration in rat lung tissue significantly enhances the thermal stability of the extracellular calcium-sensing receptor (CaSR), a mechanism potentially linked to the pathogenesis of pulmonary arterial hypertension (37). In pulmonary artery smooth muscle cells (PASMCs), the addition of exogenous phenylalanine (100 µmol/L) activates CaSR, inducing a significant increase in intracellular Ca^2+^ concentrations. Conversely, this effect is completely abolished by CaSR inhibitors or siRNA-mediated knockdown (37). These findings highlight Phe as a pivotal metabolic signaling factor involved in diverse physiological and pathological processes across various organs. Corroborating this paradigm, recent immunological studies by Kulkarni et al. identified Phe as a metabolic checkpoint that orchestrates metabolic reprogramming, specifically restricting Th2 cell proliferation and differentiation via the inhibition of STAT6 and mTOR signaling pathways (38).

Hepatic PAH activity is strictly regulated by substrate availability, cofactor cycling, and receptor-mediated phosphorylation. The entry of Phe and Leu via LAT1-CD98 allows intracellular Phe to function as an allosteric activator of PAH, while the catalytic cycle is maintained by BH4 regeneration via DHPR. Hormonal stimulation by epinephrine or glucagon activates the β-AR-cAMP-PKA axis, which phosphorylates PAH to enhance its activity and simultaneously triggers HSL-mediated lipolysis. In parallel, extracellular Phe stimulates CaSR to activate the PLC-IP_3_-PKC pathway, providing an alternative phosphorylation mechanism. Beyond catalysis, the GCH1-GFRP complex regulates BH4 synthesis for use by PAH and eNOS, while the lysosomal Sestrin2-Rag complex senses amino acid levels to modulate mTORC1 signaling.

PAH, phenylalanine hydroxylase; BH4, tetrahydrobiopterin; LAT1, L-type amino acid transporter 1; β-AR, β-adrenergic receptor; PKA, protein kinase A; HSL, hormone-sensitive lipase; CaSR, calcium-sensing receptor; PLC, phospholipase C; PKC, protein kinase C; GCH1, GTP cyclohydrolase 1; mTORC1, mammalian target of rapamycin complex 1.

## 3 Physiological functions of phenylalanine

As a fundamental substrate for protein synthesis, Phe has long been a focal point in metabolic flux studies. Historically, Moss and Schoenheimer first utilized phenylalanine tracers in murine models to elucidate the hydroxylation of Phe into Tyr (39, 40). Subsequently, the development of intravenous infusions of stable isotope-labeled Phe, combined with arteriovenous balance methods or muscle biopsy techniques, has enabled the precise quantification of whole-body and muscle protein synthesis rates (39). Beyond its role as a building block, Phe acts as a secretagogue and signaling modulator. Glucagon-like peptide-1 (GLP-1) and gastric inhibitory polypeptide (GIP), secreted by intestinal L-cells and K-cells respectively, not only augment insulin secretion (the incretin effect) but also modulate satiety (41). Recent evidence suggests that Phe, upon entering the circulation or interstitial space, binds to the basolateral CaSR, activating the PLC/DAG/PKC signaling axis and inducing intracellular Ca^2+^ release (41). This calcium signaling, coordinated with voltage-gated calcium channels, triggers the exocytosis of GLP-1 (42).Concomitantly, intracellular Phe drives leucine uptake via the LAT1 transporter. Accumulated leucine binds Sestrin2, converting Rag GTPases to their active conformation (43). Thus, Phe not only serves as a nutrient substrate, but also indirectly sustains mammalian target of rapamycin complex 1 (mTORC1) activity by maintaining high intracellular leucine concentrations. This inter-amino acid dependency ensures that anabolic programs are initiated only when the full repertoire of essential amino acids is available (44, 45). Nuclear mTOR is known to directly regulate transcription factors, epigenetic modifications, and chromatin remodeling, thereby activating the expression of genes involved in cell growth and metabolism (46). In addition to the classical Rag GTPase pathway, BCAAs activate the Rab1A-mTORC1 signaling axis to enhance the stability and transcriptional activity of the insulin transcription factor Pdx1, thereby controlling whole-body glucose homeostasis (47, 48). In contrast, Rab1A deletion selectively decreases islet β-cell number and insulin secretion (49). The above studies reveal the specific mechanism by which amino acids act as signaling molecules to regulate cellular functions through the Rab1A-mTORC1-PDX1 axis. However, as another essential amino acid, whether Phe is also involved in mTOR-mediated metabolic homeostasis needs further investigation.

Beyond protein synthesis, Phe regulates systemic metabolic homeostasis and neurotransmitter synthesis. Notably, Phe has been identified as a potent stimulator of exercise-induced fatty acid oxidation. Ueda et al. conducted a randomized double-blind crossover trial and found that acute oral supplementation of 1.5 g Phe prior to moderate-intensity aerobic exercise in healthy males led to a 12% reduction in the respiratory exchange ratio, alongside significant increases in plasma glycerol (79%) and glucagon (26%) (50).These effects may be mediated by the direct activation of α-adrenergic receptors, enhancing the efficiency of fat mobilization. Consistent with these findings, studies involving amino acid mixtures (comprising arginine, alanine, and phenylalanine) observed synergistic elevations in free fatty acids (FFAs), ketone bodies, and epinephrine levels in healthy adults (51). As a precursor to Tyr, Phe is converted via Tyr hydroxylase into catecholamines. Catecholamines can activate adipose β-adrenergic receptors and promote lipolysis (52).On the other hand, Phe stimulates the co-secretion of epinephrine and glucagon, activating adenylate cyclase and the downstream protein kinase A (PKA) pathway (52). Ultimately, PKA phosphorylates hormone-sensitive lipase, promoting its translocation from the cytosol to the surface of lipid droplets to hydrolyze triglycerides and release FFAs (50, 53). However, whether this lipolytic effect is strictly exercise-dependent or results from the synergy of specific amino acids remains a subject for further clinical investigation.

In addition, the Phe-derived metabolite Lac-Phe has emerged as a critical mediator in exercise-induced appetite suppression. High-intensity exercise stimulates significant lactate production in skeletal muscle, which is then conjugated with free Phe by the enzyme cytosolic non-specific dipeptidase 2 (CNDP2) to form Lac-Phe (reaching a 5-fold increase post-exercise) (17). Lac-Phe induced a significant reduction in food intake in obesity mice by acting on G protein-coupled receptors that control hypothalamic appetite Interestingly, this anorexigenic effect appears absent in healthy mice, suggesting that Lac-Phe may specifically target individuals with metabolic dysfunction. Clinical evidence supports a correlation between post-exercise plasma Lac-Phe increases and body fat reduction. Additionally, animal studies reveal that Phe supplementation under glycogen-depleted states enhances cerebral Tyr availability, thereby stabilizing dopaminergic neuronal activity and improving spatial memory (54). It is worth noting that previous human trials have found that fasting plasma Phe levels were significantly positively correlated with working memory scores (55). However, excessively high concentrations of Phe (>120 μmol/L) competitively inhibit tryptophan transport and interfere with serotonin synthesis, which may eventually induce anxiety risk.

## 4 Phenylalanine dysregulation in metabolic diseases: insights from epidemiologic evidence

Epidemiological evidence from numerous cross-sectional and cohort studies indicates that although the physiological fluctuations in Phe levels within the general population are subtle, their association with the risk of metabolic diseases shows high sensitivity and specificity (**Table 1**) (4, 5, 58).Metabolomics analysis showed that the difference in Phe concentration could distinguish obesity from non-obesity phenotypes (59, 60). It is well established that *PAH* gene mutations drive the excessive accumulation of Phe in the blood and brain, characterizing the autosomal recessive metabolic disorder known as PKU. Beyond the classical neurological manifestations, epidemiological evidence involving pediatric PKU populations (aged 4–15 years) indicates a heightened susceptibility to overweight and obesity compared to their healthy counterparts (61). Concomitantly, even under therapeutic dietary restriction, children with PKU exhibit significantly elevated serum triglycerides, fasting hyperinsulinemia, and a greater prevalence of MASLD relative to age-matched healthy controls. Furthermore, in the two large cohorts of SPECT-China (OR = 3.98) and UK Biobank (OR = 2.14), researchers consistently observed a significant positive correlation between serum Phe levels and the risk of MASLD (10). This suggests that abnormalities in the Phe metabolic pathway are sufficient to drive hepatic lipid deposition even in the nonobese state.

**Table 1:** Epidemiological evidence related to phenylalanine and metabolic diseases.

Diseases type	Study characteristics	Diagnostic indicators/biomarkers	Effect on metabolic diseases	References
PKU	The French SNDS database/3,549 patients with PKU	ICD-10	The risk of comorbidities (such as obesity, diabetes and hypertension) was significantly increased in PKU patients with later diagnosis	1 (12)
T2DM	The Framingham Offspring Study/2422 non-diabetic participants	Serum Phe	The adjusted risk of T2DM increased by 23% per 1 SD increase in serum Phe level	1 (5)
Diabetic retinopathy	1898 consecutive T2DM inpatients	Plasma Phe	Phe <64 µmol/L increased the risk of DR In patients with T2DM (OR = 6.01)	1 (56)
Obesity	Two cohorts/44 participants	Plasma Lac-Phe	Lac-Phe inhibits food intake, reduces adiposity and improves glucose homeostasis	1 (17)
MetS	Jordanian cohort/106 participants	Plasma Phe	Phe and its metabolite hippuric acid (144-fold increase) were the key biomarkers to distinguish patients with MetS from healthy individuals	1 (57)
MASLD	UK Biobank and SPECT-China studies/183,997 participants	Plasma Phe	Phe level was significantly positively associated with BMI, triglyceride and MASLD risk	1 (10)
Cancer	dbGaP/814 melanoma patients	*PAH* gene mutation	The frequency of *PAH* mutations was significantly positively correlated with the risk of melanoma	1 (11)

ICD-10, The International Statistical Classification of Diseases and Related Health Problems 10th Revision; SNDS, Système National des Données de Santé; Phe, phenylalanine; dbGaP, Database of Genotypes and Phenotypes; PAH, phenylalanine hydroxylase; PKU, phenylketonuria; MASLD, metabolic dysfunction-associated steatotic liver disease; MetS, metabolic syndrome; T2DM, type 2 diabetes mellitus; Lac-Phe, N-Lactoyl-Phenylalanine; OR, odds ratio; BMI, body mass index.

Phe and its downstream metabolic derivatives are also potential risk markers for metabolic diseases. Recently, Wang TJ et al. conducted liquid chromatography-tandem mass spectrometry (LC-MS) to follow a 12-year longitudinal follow-up of 2,422 normoglycemic individuals from the Framingham Offspring cohort (5). Their analysis demonstrated that each one-standard deviation (SD) increment in baseline plasma Phe was associated with a significantly higher risk of incident T2DM, yielding a multivariable-adjusted hazard ratio (HR) of 1.35 (5). We noticed that the combined amino acid metabolism score composed of isoleucine, Phe and Tyr showed effective predictive capacity, with individuals at higher levels of this score having more than 5 times the risk of future diabetes risk compared with individuals at lower levels (62). This association was further confirmed in the independent Malmö Diet and Cancer Cardiovascular Cohort and subsequently confirmed across diverse ethnicities (4). For example, a nested case-control study within the China Cardiometabolic Disease and Cancer Cohort (4C Study), involving 3,414 participants with baseline normoglycemia, showed that each SD increase in Phe correlated with a 23% increase in T2DM incidence (63). Subsequently, Mendelian randomization analysis revealed that a genetically predicted 1-SD increase in Phe levels correlates with a 60% higher incidence of T2DM (58). This evidence suggests that elevated Phe may function as a causal driver of IR rather than merely serving as a metabolic byproduct of impaired insulin signaling. Interestingly, in patients with established T2DM, abnormally low levels of plasma Phe and Tyr (< 64 μmol/L) have been linked to a sharply increased risk of diabetic retinopathy (DR), with an odds ratio (OR) of 6.01. This risk is further exacerbated (by 25.9-fold) in patients with comorbid diabetic kidney disease (56). These disparate findings reveal that the role of Phe in metabolic disease may be highly heterogeneous and context-dependent across different pathological stages.

Lac-Phe, an exercise-induced circulating metabolite first characterized by Li et al., has been identified as a potent signaling molecule that effectively suppresses food intake and promotes weight reduction in diet-induced obese mice (17). Beyond exercise physiology, Lac-Phe has become a critical plasma biomarker for mitochondrial dysfunction and septic shock. Moreover, its early-stage concentrations demonstrate superior discriminatory power for survival outcomes in septic shock patients compared to lactate alone (64). In summary, the collective epidemiological evidence indicates that Phe and its metabolic derivatives are profoundly integrated into the pathophysiological orchestration of metabolic diseases, including obesity, T2DM, and MASLD.

## 5 Pathophysiological effects of phenylalanine in metabolic diseases

### 5.1 PKU

PKU, a quintessential inborn error of metabolism, primarily stems from pathogenic variants in the hepatic *PAH* gene. Clinically, its severity is stratified based on blood Phe concentrations: severe PKU (blood Phe > 1200 µmol/L) and mild PKU (blood Phe between 600 – 1200 µmol/L) (9).Insufficient PAH activity directly obstructs the primary catabolic pathway of Phe, manifesting in three cardinal metabolic hallmarks: 1) Dietary intake of Phe cannot be hydroxylated properly, leading to its abnormal accumulation in the body, especially in the central nervous system, resulting in persistent HPA; 2) Tyr, a critical precursor for catecholamine neurotransmitters (e.g., dopamine and norepinephrine) and melanin biosynthesis, becomes relatively or absolutely deficient secondary to impaired PAH-mediated conversion; and (3) excess Phe is shunted into minor transamination pathways, generating atypical metabolites such as phenylpyruvate, phenyllactate, and phenylacetate, which may exert broad adverse effects on whole-body metabolic homeostasis (9). More than 1000 types of *PAH* gene mutations have been identified, mainly including missense mutations (58.3%), frame shift mutations (13.9%), splice site mutations (13.1%), nonsense mutations (6.9%), synonymous mutations (4.9%) and intronic or untranslated region mutations (17.9% of pathogenic mutations) (65). Mechanistically, these mutations drive a loss-of-function phenotype by abolishing catalytic activity, reducing protein stability, or disrupting the assembly and functionality of the PAH tetramer (66). Importantly, HPA can also arise from defects in the tetrahydrobiopterin (BH4) metabolic pathway or from variants in DNAJC12, which encodes a co-chaperone involved in the functional interaction network of PAH (67, 68).

The metabolic sequelae of Phe accumulation precipitate structural and functional maladaptations within the nervous system (69–75). Evidence from the *PAHenu2* mice models and clinical cohorts indicates that supra-physiological Phe levels competitively saturate the LAT1 at the blood-brain barrier (BBB) (76). Consequently, despite potential systemic Tyr supplementation, the cerebral influx of Tyr and tryptophan is attenuated due to substrate competition. Meanwhile, the constitutive expression and activity of Tyr hydroxylase (TH) and tryptophan hydroxylase 2 (TPH2) are suppressed. These enzymes represent the critical rate-limiting checkpoints in monoamine (such as dopamine and serotonin) biosynthesis (74, 75). This synergistic negative effect fundamentally impairs the biosynthetic capacity of key neurotransmitters in the brain This neurochemical deficit is a primary driver of the neuropsychiatric sequelae and executive dysfunction observed in HPA, manifesting as cognitive inflexibility, emotional dysregulation (e.g., depression and anxiety), and behavioral anomalies (66, 76–78).

Chronic HPA induces comprehensive remodeling of the epigenetic landscape within the cerebral parenchyma, characterized primarily by dysregulated DNA methylomes. Specifically, post-mortem analysis of brain from PKU patients with intellectual disabilities has revealed significant differential methylation across 158 genes (79). Among these, 100 genes exhibited hypermethylation, resulting in the inhibition of gene expression. The remaining 58 genes were hypomethylated, possibly leading to overexpression of these proteins. Notably, approximately 55 of these differentially methylated genes correspond to non-coding microRNAs (miRNAs), suggesting a sophisticated layer of post-transcriptional regulation influenced by Phe levels. Dobrowolski and colleagues further found that these miRNA-mediated epigenetic shifts translate into distinct proteomic dysregulations. Key findings include the upregulation of Synaptic Ras GTPase-activating protein (SYNGAP) and the concomitant downregulation of Gamma-aminobutyric acid type A receptor subunit alpha1 (GABRA1). While SYNGAP is an essential orchestrator of neurodevelopment and cognitive functions such as learning and memory, the depletion of GABRA1 reduces inhibitory GABAergic receptor density. This attenuation of inhibitory signaling disrupts the homeostatic excitatory/inhibitory balance, lowering the seizure threshold in affected individuals (79).

Furthermore, emerging evidence showed that elevated Phe levels drive in downstream metabolites, particularly phenylpyruvate, which acts as a potent disruptor of mitochondrial function and energy homeostasis in PKU patients (14, 15). Phenylpyruvate antagonizes the mitochondrial pyruvate carrier, restricting pyruvate influx into the mitochondrial matrix. This attenuation of substrate availability compromises the tricarboxylic acid cycle and induces dysfunction in mitochondrial respiratory chain complex I, ultimately altering oxidative phosphorylation efficiency (80–82). Simultaneously, phenylpyruvate inhibits hexokinase activity, impeding glycolytic flux and resulting in glucose utilization deficits as well as a shortage of critical intermediates required for the pentose phosphate pathway (PPP) (83–86). Animal studies have confirmed that HPA decreases the NADH/NAD^+^ ratio in the cerebral cortex and hippocampus of rats. This ratio reduction suggests a defect in mitochondrial respiratory chain complex I, manifested by reduced maximum respiratory capacity and respiratory reserve. This defect leads to reduced adenosine triphosphate (ATP) production (87). In addition to interfering with energy metabolism, metabolites like phenylpyruvate inhibit glucose-6-phosphate dehydrogenase (G6PD), the rate-limiting enzyme of the PPP. This inhibition depletes the cellular pool of NADPH, which is indispensable for maintaining glutathione in its reduced state to resist oxidative stress (80–82). Concurrently, elevated Phe levels suppress 3-hydroxy-3-methylglutaryl-coenzyme A reductase (HMGCR), the rate-limiting enzyme of the mevalonate pathway, thereby impairing the biosynthesis of ubiquinone-10 (CoQ10) (81, 88–90). CoQ10 is a key component of the mitochondrial electron transport chain (ETC), and its deficiency leads to defective ETC function (especially impaired mitochondrial complex I and III activity), which in turn increases ROS leakage and exacerbates oxidative stress (15, 90). Collectively, the synergy of impaired energy homeostasis and heightened oxidative stress accelerates neuro-functional decline, cognitive impairment, and neuropsychiatric anomalies in PKU patients (77, 91).

### 5.2 Obesity

Obesity has become a major global public health problem, and their pathophysiological processes involve complex metabolic disorders (92). Metabolomic profiling has identified amino acid dysregulation, particularly AAAs imbalances involving Phe, as a critical driver of adiposity and metabolic decline (5, 7, 8). Patients with HPA or PKU exhibit a heightened predisposition to glucolipid metabolic abnormalities and IR compared to healthy people. Clinical evidence shows that a delayed diagnosis of congenital PKU is significantly associated with an increased risk of comorbidities, including hypercholesterolemia and obesity (12). Importantly, serum Phe concentrations are significantly increased in obese pregnant women compared with their overweight pregnant counterparts and were positively correlated with pre-pregnancy BMI and Homeostatic Model Assessment of Insulin Resistance (HOMA-IR) (93). Principal component analysis (PCA) further reveals that Phe, Tyr and BCAAs constitute a distinct metabolic cluster that defines the obesity phenotype (93). This metabolic characteristic reflects a systemic shift interacting with altered gut microbiota composition (e.g., increased relative abundance of *Prevotellaceae*) and low-grade inflammation (evidenced by increased hsCRP and GlycA), which collectively exacerbate gestational IR (93).

Circulating Phe has also been identified as an important metabolic hallmark to distinguish metabolically unhealthy obesity from metabolically healthy obesity (57). Multistage metabolomic and genetic analyses have confirmed Phe as a positive diagnostic indicator for the development of obesity and MetS (57). Furthermore, Phe dysmetabolism drives a profound upregulation (approximately 144.6-fold) of downstream metabolites such as hippurate, a recognized pathological biomarker of pre-diabetes (57, 94). Similarly, it is known that under the attack of hydroxyl radicals, Phe can undergo non-enzymatic oxidation to generate non-physiological tyrosine isomers tyrosine (o-Tyr) and meta-tyrosine (m-Tyr) (95). Exogenous exposure to these isomers has been shown to impair insulin signaling and suppress glucose uptake across multiple cell lines, suggesting a novel mechanism for Phe-induced metabolic dysfunction (96).

Conversely, Phe is the indispensable biosynthetic precursor for Lac-Phe, a potent signaling molecule induced by high-intensity exercise and metformin (97, 98). Lac-Phe functions as a circulating anorexigenic metabolite that directly targets the arcuate nucleus (ARC) of the hypothalamus to suppress food intake and mitigate obesity (97, 98). For example, mice lacking CNDP2 (a key enzyme in Lac-Phe synthesis) were resistant to the weight loss and appetite suppression effects of metformin (98, 99). In contrast, Lac-Phe significantly inhibits food intake and reduces body weight in diet-induced obesity (DIO) mice but exerts no effect on lean controls, suggesting either enhanced neural sensitivity to Lac-Phe signaling under high-energy states or its role as an endogenous negative feedback mechanism (17). Despite this evidence, the precise molecular mechanisms by which Phe drives adiposity or modulates energy balance through specific signaling axes remain unclear. Future investigations must elucidate the specific pathophysiological targets and molecular drivers of Phe in the context of metabolic disorders.

### 5.3 Diabetes

Phe displays a multifaceted regulatory role in the pathogenesis of T2DM, acting as a direct etiological factor for IR and an indirect catalyst for secondary metabolic complications via its catabolites. Prospective cohort evidence from the Framingham Heart Study, utilizing LC-MS/MS analysis of 1,150 normoglycemic individuals, revealed a significant positive correlation between baseline fasting Phe levels and the future risk of T2DM (58). This association is further demonstrated by Mendelian randomization studies, which suggest a potential causal link between elevated circulating Phe and T2DM susceptibility (58). Similarly, the Phe catabolite 3-(4-hydroxyphenyl) lactate has been shown to be positively correlated with both insulin secretion and IR in 5181 Finnish men (age 57 ± 7 years, BMI 26.5 ± 3.5 kg/m²) with metabolic syndrome (100).

Recent mechanistic breakthroughs by Zhao et al. have elucidated the precise biomolecular basis for Phe-mediated impairment of insulin signaling. Within the intracellular environment, Phe serves as a substrate for the covalent modification of lysine residues (K1057 and K1079) on IRβ, which is catalyzed by phenylalanyl-tRNA synthetase (FARSA/FARSB complex) (13). This non-canonical post-translational modification, termed phenylalanylation, triggers IR-β inactivation^[14]^.Consequently, the downstream phosphorylation cascades involving insulin receptor substrate 1 (IRS1), protein kinase B (AKT), and AS160 is attenuated, effectively suppressing insulin-stimulated glucose metabolism and inducing systemic hyperglycemia (13). Consistent with these findings, transgenic mice overexpressing human FARSA (hFARSA) exhibit inhibited insulin signaling and reduced GLUT4 membrane translocation in adipose and skeletal muscle tissues (13). Importantly, this Phe-induced phenylalanylation is a reversible regulatory mechanism. Deacetylase SIRT1 can specifically remove the F-K1057/1079 modification on IR-β, thereby restoring receptor sensitivity and improving glucose tolerance (13). In parallel, dietary Phe excess in finishing pigs has been observed to downregulate the expression of the PI3K/Akt pathway in the pancreas and liver, while concurrently inhibiting β-cell proliferation and insulin secretory capacity, collectively exacerbating glucose dyshomeostasis (101).

In addition to its negative effects on insulin sensitivity, Phe has been identified as promoting and maintaining chronic low-grade inflammation in diabetic complications (102). In diabetic foot ulcers (DFU), abnormally elevated Phe leads to the accumulation of its downstream metabolite phenylpyruvate (PPA). PPA enters macrophages via CD36-mediated uptake and subsequently inhibits the activity of palmitoyl-protein thioesterase 1 (PPT1, a key lysosomal enzyme). The suppression of PPT1 induces the aberrant palmitoylation of the NOD-like receptor thermal protein domain associated protein 3(NLRP3) inflammasome, which improves protein stability, eludes lysosomal degradation, and promotes pathological hyperactivation of the inflammasome (102). This mechanism triggers the excessive release of pro-inflammatory cytokines (such as interleukin-1β), thereby exacerbating localized inflammation and impeding cutaneous wound healing in DFU patients (102). Clinical observational studies have reported a significantly positive correlation between supraphysiological Phe levels and the increased risk of T2DM and its macrovascular complications (such as cardiovascular disease) (56, 57, 103–105). Interestingly, another cross-sectional survey revealed a paradoxical association: abnormally low plasma Phe levels significantly increase the risk of diabetic retinopathy in T2DM patients. This risk is likely mediated by the suppression of Tyr and downstream dopamine synthesis, with a significant additive effect observed (56). In conclusion, these results support the view that deviation from Phe metabolic homeostasis, whether pathological elevation or abnormal depletion, tends to accelerate the progression of T2DM and its associated systemic complications.

### 5.4 MASLD

The global prevalence of MASLD has reached 24% and is a critical driver of the progression of cirrhosis and hepatocellular carcinoma (106, 107). Multiple studies have found a significant decline in hepatic PAH enzymatic activity, accompanied by pathologically elevated circulating Phe levels, in both patients with MASLD and animal models such as DIO mice. HPA has been demonstrated not only to correlate significantly with hepatic fat fraction but also to exhibit robust positive associations with BMI, serum triglycerides, and hepatocellular injury (10). These observations suggest that Phe accumulation occurs in tandem with hepatic damage and systemic dyslipidemia, although the causal relationship remains to be fully elucidated. Recently, Sun et al. demonstrated that high Phe concentrations downregulate the expression of B-cell lymphoma 2 (Bcl-2)/adenovirus E1B 19-kDa interacting protein 3 (BNIP3) in HepG2 cells and rat livers. This downregulation suppresses mitophagy/autophagy and promotes mitochondrial dysfunction, ultimately leading to exacerbated hepatic lipid accretion (10).

Phenylacetic acid (PAA), a metabolite of Phe, is also an important pathogenic factor. PAA treatment in primary human hepatocytes significantly reduces Akt phosphorylation, thereby inducing hepatocellular insulin resistance (108). Importantly, excessive PAA can be further metabolized by the gut-liver axis into phenylacetylglutamine (PAGln). PAGln increases B-type natriuretic peptide and attenuates cardiomyocyte contraction both *in vitro* and *in vivo*, which may increase cardiovascular risk (development of complications of heart failure) in patients with liver dysfunction (109). Similarly, the Phe derivative N-acetyl-phenylalanine (NAPA) has recently been reported to accelerate MASLD progression. Mechanistically, NAPA promotes hepatic steatosis by disrupting endoplasmic reticulum (ER)-mitochondria calcium coupling and impairing mitochondrial fatty acid oxidation (110). In summary, the circulating Phe pool and its downstream metabolic derivatives represent key points in the pathogenesis and progression of MASLD. These molecules have significant potential as clinical therapeutic targets, and future research is warranted to clarify their translational value for targeted interventions.

### 5.5 Cancer

Numerous studies have confirmed that abnormal Phe metabolism is closely related to tumorigenesis, progression and complications of cancer. A landmark genetic analysis has characterized the direct link between *PAH* deficiency and specific oncogenic risks. By performing germline whole-exome sequencing on 814 melanoma patients across three public databases, researchers identified a 3.56% carrier frequency for PKU/HPA-associated pathogenic *PAH* variants, which was significantly higher than the 1.76% frequency observed in matched healthy populations, representing an approximately 2.02-fold enrichment (11). The systemic Phe elevation and subsequent Phe/Tyr ratio imbalance caused by impaired PAH activity may suppress melanogenesis and promote the malignant transformation of melanocytes via activation of the mitogen-activated protein kinase (MAPK) pathway (11). Furthermore, Kink et al. demonstrated that breast cancer patients with comorbid depression exhibit a significantly higher circulating Phe/Tyr ratio compared to healthy controls, non-depressed breast cancer patients, or non-cancerous individuals with depression. Both cancer-associated anemia and diminished quality of life were strongly associated with this metabolic derangement in this patient cohort (111). The underlying pathophysiology is primarily driven by tumor-associated inflammation, which interferes with the BH4 pathway. Chronic release of pro-inflammatory cytokines, notably interferon-γ (IFN-γ), augments reactive oxygen species (ROS) production and continuously depletes BH4 bioavailability (111). Given that BH4 is an obligatory cofactor for both PAH and Tyr hydroxylase (catalyzing Tyr conversion to the dopamine precursor L-DOPA), BH4 depletion simultaneously triggers Phe accumulation (elevating the Phe/Tyr ratio) and hinders the synthesis of monoaminergic neurotransmitters such as dopamine, thereby precipitating depressive symptoms (111).

During tumor cell proliferation, Phe is not only a nutrient substrate, but also a key signaling molecule involved in metabolic reprogramming. LAT1 is ubiquitously overexpressed across a spectrum of malignancies, including lung, breast, glioma, and hepatocellular carcinoma (112). Specifically, in triple-negative breast cancer and gastric cancer cells, LAT1-mediated Phe uptake is necessary for the constitutive activation of the mechanistic target of rapamycin complex 1 (mTORC1) (112). Treatment with LAT1-specific inhibitors (e.g., JPH203) or pan-L-type amino acid transporter inhibitors (e.g., BCH) to reduce intracellular Phe levels has been shown to attenuate the mTORC1/p70S6K signaling cascade, subsequently inducing cell cycle arrest and apoptosis (112). Furthermore, the metabolic status of Phe is also associated with the pathogenesis of cancer cachexia. In the C26 colon carcinoma mice model, pathologically elevated serum Phe concentrations show a significantly negative correlation with body weight, implying that circulating Phe levels may serve as a viable biomarker for predicting tumor progression and evaluating the severity of cachexia (113).

In addition to participating in cellular anabolism through transport proteins, Phe also modulates the tumor immune microenvironment through its enzymatic derivatives. For instance, Interleukin-4-induced gene 1 (IL4I1), a secreted L-amino acid oxidase, catalyzes the conversion of Phe into phenylpyruvate and hydrogen peroxide (H_2_O_2_) (114).The resultant H_2_O_2_ induces localized oxidative stress, which significantly suppresses the proliferation of CD3-stimulated CD4^+^ and CD8^+^ T cells, thereby impairing anti-tumor capacity (114).On the other hand, subsequently damages their cytotoxic phenylpyruvate is an endogenous ligand for the aryl hydrocarbon receptor (AHR) (115).AHR activation by phenylpyruvate promoted the differentiation of CD4^+^ T cells into regulatory T cells (Tregs) while inhibiting pro-inflammatory Th17 cell differentiation (114). Notably, overexpression of IL4I1 in colorectal cancer cells has been shown to enhance AHR nuclear translocation, leading to the upregulation of genes associated with CD8^+^ T cell exhaustion (such as *PD-1* and *TIM-3*), thereby impairing their cytotoxic activity (115). These mechanisms define Phe as a critical metabolite bridging metabolic reprogramming and immune checkpoint regulation. Interestingly, although host Phe metabolism promotes tumor progression, specific gut microbiota-derived metabolites exhibit anti-tumor potential. Ding et al. discovered that the gut commensal *Faecalibacterium prausnitzii* activates Phe metabolism to produce PAGln, which significantly inhibits the growth of ovarian and prostate cancers (116). Mechanistically, PAGln upregulates Cyclin G2 (CCNG2) expression, which in turn antagonizes the Wnt/β-catenin signaling pathway (116).This cascade effectively suppresses the proliferation, migration, and invasion of prostate cancer cells (117).Furthermore, PAGln has been found to induce intratumoral ferroptosis by downregulating HO-1 expression, increasing lipid peroxidation, and depleting intracellular glutathione (116).These findings highlight that modulating the gut microbiota or supplementing specific Phe metabolites may serve as a novel therapeutic strategy for inducing ferroptosis in oncological settings.

**Figure 2:**
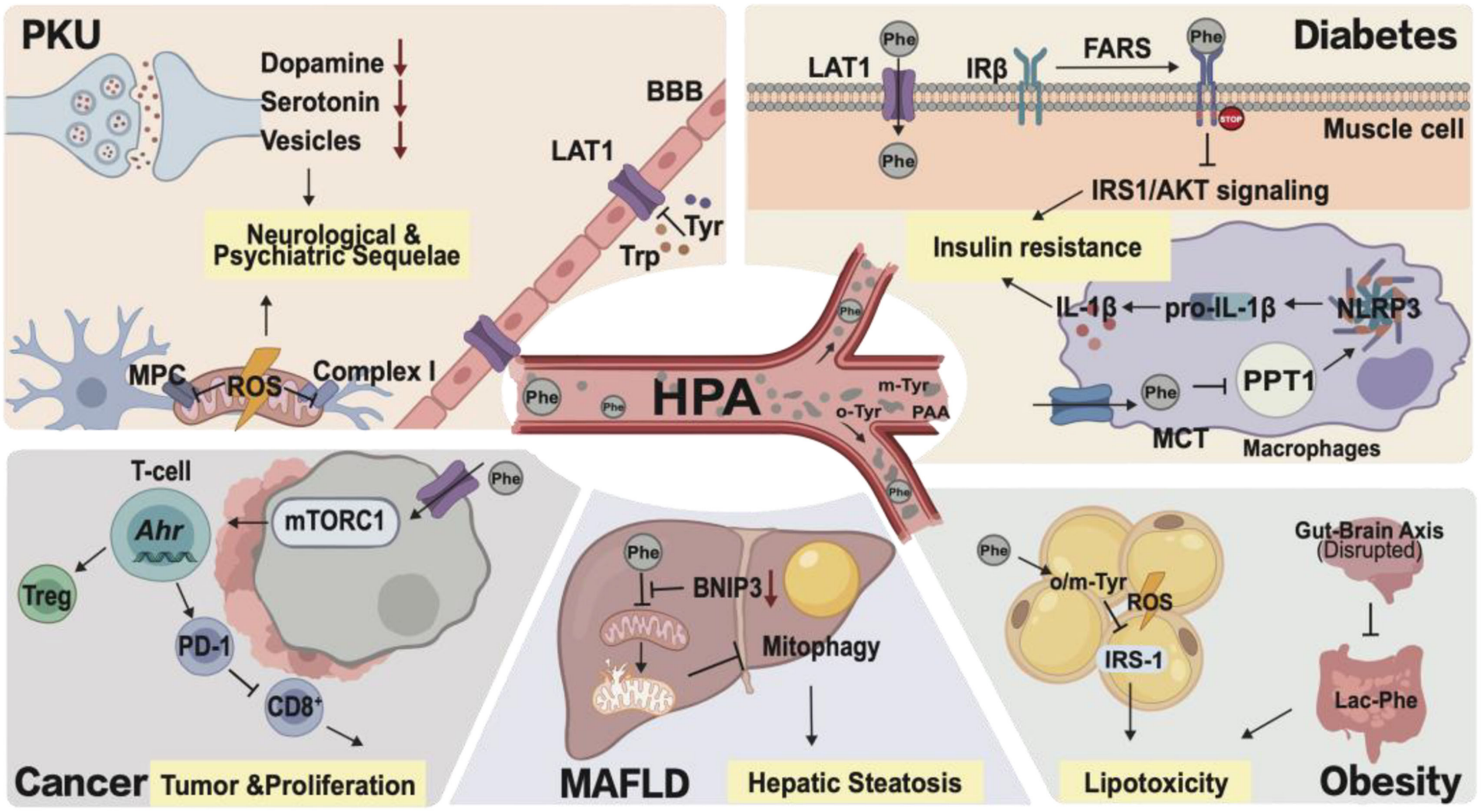
The systemic pathological network of phenylalanine metabolism.

Despite initial progress in the mechanisms of abnormal Phe levels in metabolic diseases (**Figure 2**), the temporal dynamics of Phe metabolism dysfunction and its heterogeneity across various malignancies remain to be explored. Future research should use patient-derived organoids and prospective clinical cohorts to verify the therapeutic potential of targeting Phe.

HPA drives multi-organ dysfunction via distinct molecular pathways. Centrally, Phe accumulation disrupts LAT1 transport and mitochondrial function, causing neurological deficits. Peripherally, Phe induces insulin resistance via FARS-mediated IRβ modification and NLRP3 hyperactivation, while its oxidative isomers (o/m-Tyr) and disrupted Lac-Phe signaling exacerbate lipotoxicity and obesity. In cancer, Phe promotes cellular proliferation via the LAT1-mTORC1 axis and facilitates immune evasion through AhR-mediated T-cell exhaustion. HPA, hyperphenylalaninemia; PKU, phenylketonuria; BBB, blood-brain barrier; LAT1, L-type amino acid transporter 1; ROS, reactive oxygen species; FARS, phenylalanyl-tRNA synthetase; IRβ, insulin receptor beta; PAA, phenylacetic acid; PPT1, palmitoyl-protein thioesterase 1; NLRP3, NLR family pyrin domain containing 3; MAFLD, metabolic dysfunction-associated fatty liver disease; MPC, mitochondrial pyruvate carrier; BNIP3, BCL2 interacting protein 3; o/m-Tyr, ortho/meta-tyrosine; Lac-Phe, N-lactoyl-phenylalanine; mTORC1, mammalian target of rapamycin complex 1; AhR, aryl hydrocarbon receptor; Treg, regulatory T cell; PD-1, programmed cell death protein 1.

## 6 Therapeutic strategies targeting the phenylalanine metabolic network

**Figure 3:**
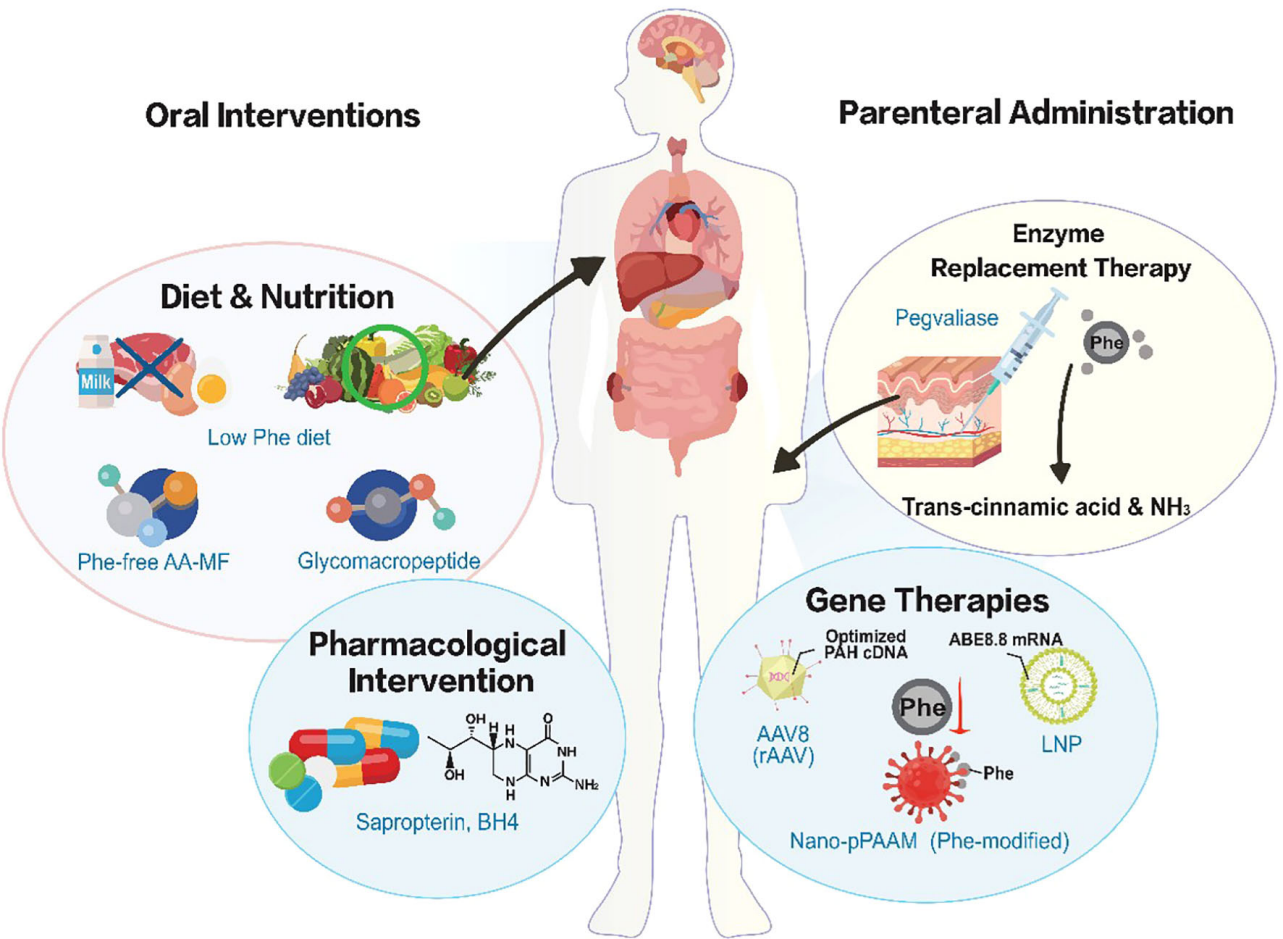
Current and emerging therapeutic strategies for PKU. Management strategies are categorized by administration route. Oral interventions include dietary restriction supplemented with amino acid medical foods (AA-MF) or glycomacropeptide (GMP), and pharmacological support via sapropterin (BH4). Parenteral therapies feature enzyme replacement with Pegvaliase. Advanced approaches utilize AAV8 vectors for gene restoration, lipid nanoparticles (LNPs) for base editing (ABE8.8), and Phe-surface-modified nanomedicines (Nano-pPAAM) for targeted therapy. PKU, phenylketonuria; AA-MF, amino acid medical foods; GMP, glycomacropeptide; BH4, tetrahydrobiopterin; AAV, adeno-associated virus; LNP, lipid nanoparticle; ABE, adenine base editor; Nano-pPAAM, Phe-modified porous amino acid-mimetic nanomedicine.

The pathological implications of dysregulated Phe metabolism transcend the classical manifestations of PKU, intersecting with prevalent metabolic disorders including obesity, T2DM, and malignancy. Accordingly, clinical therapeutic strategies are gradually transitioning from traditional strict dietary restriction to personalized and multimodal interventions including nutrition, pharmacology and enzyme replacement therapy (**Table 2** and **Figure 3**).

**Table 2:** Current potential therapeutic strategies.

Diseases	Therapeutic strategies	Treatment methods	References
PKU	① Diet therapy ② Enzyme replacement therapy ③ Gene therapy ④ Pharmacological therapy	① Low Phe diet; GMP ② Pegvaliase/PAL therapy ③ Repair *PAH* mutant genes ④ Sepiapterin/Sapropter/SYNB1618	1 (118, 141, 150–152)
Obesity	① Diet therapy ② Gene therapy	① Moderate low phenylalanine diet ② SIRT1 activator	1 (13, 153)
T2DM	**① **Gene therapy **② **Pharmacological therapy **③ **Diet therapy	① SIRT1 activator ② Metformin/Phenylpropanol ③ Moderate low Phe diet	1 (13, 153, 154)
MASLD	① Pharmacological therapy ② Diet therapy	① Rapamycin ② Moderate low Phe diet/Gut microbiota regulation	1 (10, 153–155)
Cancer	① Enzyme replacement therapy ② Pharmacological therapy ③ Radiotherapy	① PAL therapy ② LAT1 inhibitors(JPH203) ③ Boron neutron capture therapy	1 (97, 98, 155–157)

GMP, Glycomacropeptide; PAL, phenylalanine ammonia-lyase; T2DM, type 2 diabetes mellitus; PKU, phenylketonuria; MASLD, metabolic dysfunction-associated steatotic liver disease; SIRT1, Sirtuin 1.

### 6.1 Nutritional intervention

Lifelong adherence to a stringent low-Phe diet remains the cornerstone of effective PKU management, involving the restriction of natural protein intake supplemented with Phe-free amino acid formulas (118). The primary therapeutic objective is to maintain circulating Phe levels within a targeted range (< 360 μmol/L) to minimize neurocognitive impairment (9, 119). Studies have shown that achieving and maintaining this target can reverse the competitive inhibition of LNAA transporters at the BBB caused by hyperphenylalaninemia. This restoration of neurotransmitter precursor transport contributes to ameliorate executive dysfunction and affective disorders in PKU patients (120). However, the long-term efficacy of dietary therapy is often restricted by poor adherence, monotony, and micronutrient deficiencies (including calcium, iron, and essential vitamins) (118). Researchers have further proposed a vicious cycle: the more patients deviate from dietary restrictions due to low adherence, the higher the plasma Phe level. This elevation directly impaired the executive function of the prefrontal cortex, which is responsible for impulse control and decision making (121, 122). This neurocognitive decline in turn undermines the patient’s capacity to strictly follow the diet plan, resulting in further increases in blood Phe (123–125). To address this predicament, Glycomacropeptide (GMP), a low-Phe peptide derived from cheese whey, has been integrated into clinical practice (126). GMP significantly attenuates postprandial circulating ghrelin levels, thereby facilitating appetite regulation (127). This is essential to prevent obesity caused by excessive intake of low protein and high starchy foods in PKU patients (127). Furthermore, GMP has been shown to improve emotion by increasing Tyr availability. And because of its high concentration of BCAAs, it can promote bone mass accumulation, thereby reducing the long-term risk of osteoporosis in PKU. Nevertheless, clinical utilization of GMP-MF must account for the residual Phe content (approximately 1.8 mg/g protein) within the total daily allowance and ensure adequate supplementation of naturally deficient nutrients (128). As mentioned above, in the context of obesity or a high-protein diet, FARS mediates the phenylalanylation of the lysine residues of the IRβ, which in turn elicits insulin resistance (13). Therefore, dietary structure optimization, such as the adoption of plant-based diets characterized by lower Phe content and its bioavailability, was potentially efficacious interventions. Previous studies have demonstrated that vegetarian or vegan diets can significantly reduce plasma Phe and improve management of T2DM (23, 129). This adjustment of the amino acid profile is generally significantly correlated with reductions in body weight and improvements in fasting insulin sensitivity (130, 131).

### 6.2 Pharmacological therapy

Conventional sapropterin dihydrochloride (Sapropterin/Kuvan) is a synthetic analog of BH4, the essential cofactor for PAH. It functions as a pharmacological chaperone by stabilizing the conformation of mutant PAH, thereby enhancing residual enzymatic activity (18, 19). Clinical evidence indicates that therapeutic responsiveness to sapropterin is limited to approximately 20–30% of the PKU population, predominantly involving those with mild-to-moderate phenotypes (132). Long-term management combining sapropterin with dietary Phe restriction significantly attenuates blood Phe concentrations, which can further permit dietary liberalization and improve the patient’s quality of life (133, 134). However, Sapropterin is not clinically effective for patients with classical PKU (accounting for over 70% of the total PKU patient population) who have complete loss of PAH enzyme activity due to missense or nonsense mutations (132). On the other hand, sepiapterin has recently been approved by the U.S. Food and Drug Administration (FDA) for clinical use in the treatment of PKU (135). As a synthetic oral precursor of BH4, sepiapterin is internalized via active transport and undergoes a rapid intracellular conversion into BH4 (136). Notably, sepiapterin has been shown to be more effective than exogenous sapropterin in increasing bioavailable BH4 within diverse target tissues (137). The safety, pharmacokinetic, and therapeutic efficacy of sepiapterin have been systematically evaluated in Phase I, II, and III clinical trials (137–139). These studies demonstrate that sepiapterin intervention induces a significant 63% reduction in blood Phe levels across pediatric and adult cohorts, with 84% of participants achieving the target range (120–360 μmol/L) recommended by the American College of Medical Genetics and Genomics (ACMG) (136). Furthermore, sephience is well tolerated, with no serious adverse events or drug discontinuation reported, and is suitable for PKU patients who exhibit a poor response to current therapies or struggle to adhere to long-term strict dietary control (140).

### 6.3 Enzyme replacement therapy

To avoid the limitations inherent to dietary restriction alone or pharmacologic therapies, PAH-independent enzyme-replacement therapy (ERT) was developed (66). Pegvaliase (Palynziq) is a pegylated recombinant Phe ammonia lyase administered via subcutaneous injection (20). It metabolizes systemic Phe into trans-cinnamic acid and ammonia, which are subsequently processed by the liver and excreted (66). Analysis of Phase III clinical trials and subsequent real-world evidence demonstrates that patients in the pegvaliase-treated cohorts achieved significant and sustained reductions in blood Phe levels throughout 12, 24, and 36 month follow-up periods compared to conventional dietary management (20, 141, 142). Importantly, most responders achieved completely discontinued medical food intake (restoring a normal diet) during pegvaliase therapy. Over 60% of patients attained plasma Phe levels below 360 μmol/L, with 78.1% of this subgroup reaching physiologically normal levels (≤ 120 μmol/L) (141, 142). However, there are concerns about the safety of Pegvaliase in clinical applications. Nearly 100% of patients experience injection site reactions (such as erythema and pain) and arthralgia during the early stages of treatment. More significantly, the induction phase is associated with a risk of anaphylaxis, necessitating medical supervision during initial dosing (66). Consequently, pegvaliase is currently indicated primarily for adult PKU patients who exhibit inadequate metabolic control despite dietary restriction or sepiapterin therapy (143). In addition, enzyme therapy has also been attempted for cancer treatment by taking advantage of the high dependence of tumor cells on external Phe. Pegylated phenylalanine ammonia lyase causes amino acid starvation in tumor cells by depleting circulating Phe pools, leading to impaired protein synthesis and cell cycle arrest, ultimately inducing autophagic cell death (19). However, the current clinical immunogenicity, enzyme production and drug delivery methods need to be further optimized.

### 6.4 Gene therapy

Recombinant adeno-associated virus (rAAV)-mediated gene therapy aims to deliver functional copies of the *PAH* gene into the hepatocyte nucleus. However, this approach remains in the preclinical or early clinical stages. For instance, in the *Pah^enu2^* mouse model, hepatic delivery of AAV8 harboring codon-optimized *PAH* cDNA restored blood Phe levels to near-physiological levels for over a year (144, 145). Nevertheless, rAAV-based therapies face practical challenges, particularly host immune responses directed against the viral capsid or the transgene product, which may reduce efficacy or impede re-administration (132, 146). Meanwhile, *in situ* genomic repair utilizing CRISPR technology has emerged as a potential therapeutic prospect. Lipid nanoparticles (LNPs), widely employed as non-viral delivery platforms, can facilitate the systemic delivery of mRNA or gene-editing machinery to various organs (147). ABE8.8 mRNA encapsulated in LNPs effectively targets and rescues pathogenic *PAH* gene in hepatocytes (148). It is worth noting that blood Phe levels were normalized within 48 hours and were maintained for over 24 weeks, demonstrating the enduring therapeutic potential of base editing (132). And translation of this strategy to mice and non-human primates resulted in no apparent hepatotoxicity or off-target mutations (132).

In the context of oncology, a Nanoscopic phenylalanine Porous Amino Acid Mimic (Nano-pPAAM) has been reported as a new nanotherapeutic agent with intrinsic anticancer activity and high selectivity for malignancy (149). By disguising itself as nutrients, Nano-pPAAM is actively and efficiently internalized by the tumor via LAT1. Upon internalization, Nano-pPAAM triggers excessive ROS production, subsequently activating both extrinsic and intrinsic apoptotic pathways (149). In NOD scid gamma (NSG) mice bearing MDA-MB-231 xenograft, this approach achieved a 60% overall inhibition of tumor growth in the absence of exogenous drugs or external intervention (149).

## 7 Conclusion

Phe has evolved from a conventional substrate for protein synthesis to a critical signaling factor that coordinates metabolic homeostasis. By integrating epidemiological and multi-omics evidence, this review elucidates the profound impact of increasing circulating Phe levels across a spectrum of metabolic disorders, including obesity, T2DM and cancer. In particular, the pathogenicity of Phe transcends its classical neurotoxicity observed in PKU. It directly impairs the insulin signaling axis through FARS-mediated phenylalanylation of the IR-β (158). Furthermore, Phe and its downstream catabolites (e.g., phenylpyruvate) exacerbate chronic inflammation and energetic dysregulation by impairing mitochondrial function and activating the NLRP3 inflammasome. These interactions may contribute to a complex pathophysiological feedback loop associated with the progression of metabolic diseases (39, 102).

From classic dietary restriction to gene editing, the clinical management of Phe metabolic disorders is undergoing a technological transformation. Currently, diversified therapeutic strategies include the novel oral cofactor sepiapterin, recombinant enzyme replacement therapy, and LNP-mediated CRISPR/Cas9 genomic repair, all of which have demonstrated significant clinical potential (20, 135, 147). However, ensuring the continuity, efficacy and safety of the treatment strategy remains a formidable challenge, particularly in the context of multiple comorbidities (119). Future advancements may utilize nanotechnology for precision drug delivery. For instance, biomimetic nanoparticles encapsulating the *PAH* genes could avoid the adverse immune responses inherent to AAV-mediated delivery (159). The integration of artificial intelligence to construct personalized, dynamic amino acid metabolic scoring models may facilitate precision nutritional guidance for patients with metabolic diseases (160, 161). Furthermore, future researchers should clarify the receptor signaling mechanisms of endogenous molecules, to develop non-Phe-dependent analogs for appetite suppression. Integrating the modulation of Lac-Phe signaling with established metabolic interventions, such as GLP-1 receptor agonists (GLP-1RAs), may yield synergistic efficacy in promoting weight loss and improving glucose homeostasis (162, 163).
